# Comparison of sequential versus pre mixed administration of intrathecal fentanyl with hyperbaric bupivacaine for patients undergoing elective Caesarean section at Zewditu memorial referral hospital: A prospective cohort study

**DOI:** 10.1016/j.amsu.2022.103313

**Published:** 2022-01-27

**Authors:** Animut Tilahun Chekole, Adugna Aregawi Kassa, Senait Aweke Yadeta, Habtu Adane Aytolign

**Affiliations:** a Department of Anesthesia^,^ Hiwot Fana Comprehensive Specialized Hospital, Harar, Ethiopia; b Department of Anesthesia, College of Health Science and Medicine, Addis Ababa University, Addis Ababa, Ethiopia; c Department of Anesthesia College of Medicine and Health Science, University of Gondar, Gondar, Ethiopia

**Keywords:** Hyperbaric bupivacaine, Fentanyl, Spinal anesthesia, Pre mixed, Sequential, Cesarean section

## Abstract

**Background:**

Spinal anesthesia (SA) is the method of choice for surgery below umbilicus like elective cesarean section. However, Spinal anesthesia is associated with hypotension and limited analgesia duration. To minimize those complications adding opioids like fentanyl either sequentially with separate syringe or pre mixed with local anesthetics become common practice.

**Objective:**

To compare the hemodynamic and analgesic effect of sequential versus pre mixed injection of intrathecal fentanyl with hyperbaric bupivacaine for patients who underwent elective CS under Spinal anesthesia.

**Method:**

A prospective cohort study was performed on parturient who undergone elective cesarean section from 01 January 2020 to 30 March 2020. The decision to give either sequential or premixed drug was based on the responsible anesthetists. Sixty-six American society of Anesthesiologist Ⅱ age ≥18 was recruited. Those who received sequentially were grouped as (S- group) and those who had received pre mixed technique were grouped as (M-group). Data were entered into Epi Info version 7.0 and transported into SPSS Version 22 for analysis. Based on normality assumption, analysis was done by independent *t*-test for normally distributed data. Whereas Mann –Whitney *U* test for non-normally distributed data and x2 (Chi-square) test for categorical variable. P-value <0.05 was considered as statistically significant.

**Result:**

Significant reduction in intra operative mean arterial blood pressure was seen in premixed group compared to Sequential group until 15th minute immediately after spinal anesthesia. Thus, the incidence of hypotension was higher in M − group compared to S- group, (p < 0.05). The median Postoperative pain VAS score was significantly lower in *the S* - *group* compared to M − group *of* 4th, 5th and 6th hr. The mean time for 1st rescue analgesic request time was prolonged in *the* S - *group* compared to M − group (287.909 ± 15.255 vs. 261.39 ± 25.378) min respectively (p < 0.001).

**Conclusion:**

The Sequential intrathecal injection of fentanyl and hyperbaric bupivacaine provided significant improvement in the blood pressure stability and of sensory and motor block compared to premixed groups.

## Highlights

• Spinal anesthesia is associated with hypotension and limited analgesia duration.
• To minimize those complications adding opioids like fentanyl either pre mixed with local anesthetics or in separate syringe become common practice.
• Sequential intrathecal injection of fentanyl and hyperbaric bupivacaine provided significant improvement in the blood pressure stability and of sensory and motor block compared to premixed groups.
• Longer time to first analgesia request was seen in sequential group compared with premixing group.
• There was significant lower total tramadol consumption in sequential compared with premixing group but comparable total diclofenac consumption.

## 1 Introduction

Caesarean section (CS) is lifesaving procedure when there is both or either maternal and fetal problem. The rate of CS increases dramatically from time to time [1]. Regional anesthesia techniques are highly preferred for CS compared to general anesthesia [2,3]. Spinal is a reversible nerve transmission interruption due to injection of local anesthetics in sub arachnoid space. Local anesthetic agents for spinal can be either isobaric or hyperbaric. Baricity of the drug represents the ratio of the specific density of anesthetics and cerebrospinal fluid (CSF). hyperbaric is heavier than the CSF which is gravity dependent [5].

Even though spinal anesthesia is preferable for CS, it is associated with different complications among these hypotension is the most common one [6]. This is mostly because of sympathetic blockade leading to lower extremity peripheral vasodilatation and pooling of blood in dilated vascular area causing decrease of venous return and cardiac output [4]. This problem is profound in pregnancy due to hormonal changes and gravid uterus compresses great vessels against the bodies of the lumbar vertebra leading to a compromised uteroplacental blood flow [2,7]. Moreover, post cesarean section pain is also another challenge and spinal anesthesia has limited analgesia duration [8].

Many trials have been conducted to prevent hypotension immediately after spinal injection [9,10]. Fluid therapy, but still there is a controversy about the administration of crystalloids or colloids; preloading or co loading [11]. The use of vasopressor, the choice and mode of administration as either bolus or infusion is still a matter of debate [12,13].

There are also many posts cesarean section pain management options including neuraxial (epidural and spinal with adjuvants), Bilateral Transversus Abdominis Plane (TAP), bilateral Ilioinguinal-Ili hypogastric Nerve Block, wound infiltration and multimodal analgesia [14]. Each of them has an advantage and disadvantage.

The addition of adjuvants like opioids, clonidine and dexmedetomidine have been reduced in hemodynamic change and improving analgesic quality at the same time [[15], [16], [17]].

Intrathecal fentanyl enhances analgesia with bupivacaine and achieve successful subarachnoid block by acting on mu-receptor found in spinal cord and resulted in improved quality and prolonged analgesia. In addition, fentanyl acts on the afferent nociceptive path so it causes less adverse hemodynamic effects [18,19].

However, the effect of sequential or pre mixed fentanyl with hyperbaric bupivacaine is not clearly known [18]. There are different controversy regarding sequential and premixing administration of opioids with bupivacaine for spinal anesthesia [5,20,21]. Furthermore, sequential vs. pre mixed fentanyl with bupivacaine are commonly practiced spinal anesthesia techniques in the study area, but their effect was not known yet. Thus, the aim of this study was to compare the hemodynamic and analgesic effect of fentanyl with hyperbaric bupivacaine sequential with two syringe techniques compared to pre mixed with one syringe injection of spinal anesthesia for patients undergoing elective cesarean section at referral hospital.

## 2 Methodology

### 2.1 Study design, period and study area

An institutional based prospective cohort study was conducted from 01 January 2020 to 30 March 2020. The study was conducted in referral hospital. The Hospital is operated by the ministry of health which has a total of 128 beds out of these 46 beds are for obstetrics, gynecology and postnatal ward. The research was registered with a research registry unique identifying number of researchregistry7457. This study has also reported in line with STROCSS 2021 criteria [22].

### 2.2 Population

All mothers who underwent elective cesarean section under spinal anesthesia were Source of population. Whereas all mothers who underwent elective cesarean section under spinal anesthesia with premixed or sequential fentanyl with hyperbaric bupivacaine were Study population.

### 2.3 Eligibility criteria

All ASA physical status Ⅱ parturient who were undergoing elective cesarean section delivery under spinal anesthesia either sequential vs. premixed fentanyl combined with hyperbaric bupivacaine. Height between 145&175 cm as height affects spinal anesthesia and Body mass index (BMI) (as obesity compromise hemodynamic status) between 18.5 and 30 kg/m^2^ were included. While pre-operation hypotension and bradycardia, Preeclampsia, Multiple pregnancy and macrosomia, Complete or partial failed spinal, Patients taking other than 10 mg bupivacaine and 25 μg fentanyl, Patients taking isobaric bupivacaine for spinal and Patients having regional nerve block other than SA were also excluded.

### 2.4 Study variables

#### 2.4.1 Independent variables

Socio-demographic variables (Age, weight, height and BMI), parity, position, blood loss, amount of intra operative fluid intake, duration of surgery, base line MAP and HR.

#### 2.4.2 Dependent variables

Change in intra and post-operative Mean arterial blood pressure and mean heart rate.

Post-operative pain VAS score.

First rescue analgesic request time.

### 2.5 Operational definition

**Hypotension**: is when Systolic blood pressure of below 90 mmHg or MAP less than 70 mmHg on one or more observation [19].

**Bradycardia**: is when heart rate less than 60 beats/minutes on one or more observation [16].

**Respiratory depression**: is respiratory rate less than 10 breaths per minute or oxygen saturation less than 90% [19].

**Pruritus**: any scratch or itching complained by the patient or visible rash after SA.

**Fig. 1:**
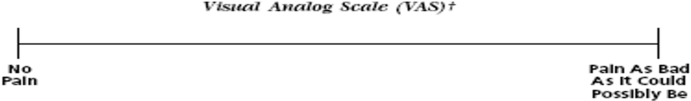
VAS instrument adopted from the National Initiative on Pain Control™ (NIPC™). 0 cm = no pain 4–6 cm = moderate pain. 1–3 cm = mild pain 7–10 cm = sever pain.

**VAS**: A visual analogue scale which is a method of pain assessment determined by the patient making a mark (/) of their pain intensity on a line which is 10 cm long (34) (Fig. 1).

**Nausea**: is subjectively unpleasant sensation associated with awareness tee urge to vomit.

**Total analgesia consumption**: the total amount of analgesic the patient was given within 24 h after SA.

**Onset of sensory block**: time elapsed from the end of spinal injection to absence of pinprick sensation at T10 dermatome.

**Time to first analgesic request**: is a time in minute measured from the end of spinal anesthesia procedure to the time where patient request analgesics.

**ASA Ⅱ**: pregnant mothers without any co-existing illness comes for cesarean section.

**Modified Bromage scale**, 0: No motor block, 1: Unable to raise an extended leg (able to flex the knee), 2: Unable to flex the knee (able to move the foot only), 3: Unable to flex the ankle (unable to move the foot or knee) [23].

### 2.6 Sample size and sampling technique

#### 2.6.1 Sample size determination

The mean and standard deviation of the sensory regression with sequential and premixed fentanyl and bupivacaine after spinal anesthesia was 93.5 ± 10.0 and 85.5 ± 11.5 respectively [21]. Assuming 1:1 ratio in the two groups with the power of 80% and the level of significance α = 0.05, then the sample size was calculated in the following formula;

2) N= (α +p) ^2^ (sd1^2^+sd2^2^) / (μ1-μ ^2^

–85.5) N= (0.84 + 1.96) ^2^ (10^2^+11.5^2^) / (93.5 ^2^

29 N = 28.4 =

When 15% of contingency is included for dropouts, approximately 33 patients per group and the total sample size was = 66.

#### 2.6.2 Sampling technique

The assigned anesthetists in the operation theater were responsible to give either sequential or pre mixed spinal anesthesia based on their interest. The type of spinal anesthesia (sequential or premixed) was not clearly recorded except specific code on the patient chart by the responsible anesthetist. only investigator knew that specific code by communicating with the responsible anesthetists. Thus, the data collectors were blind about the type of SA that the patient was given. Parturient were selected consecutively till the required sample sizes were achieved in each group. Parturient who received sequential (S) fentanyl and bupivacaine considered as an exposure group while those who received premixed (M) fentanyl and bupivacaine were considered as a control group.

### 2.7 Anesthesia standard protocol

Anesthesia management was in standard form according to the hospital protocol. It is common practice to administer Metoclopramide premedication 10 mg IV and Pre loading all parturient with 1000 ml normal saline. All medications including for general anesthesia were prepared before insertion of the spinal needle and standard monitoring such as noninvasive blood pressure (NIBP), pulse oximetry and ECG were attached and baseline vital sign was recorded on the anesthesia recording sheet by the responsible anesthetists. After the site has cleaned with antiseptic technique, the spinal anesthesia was given by the responsible anesthetist in sitting position either sequentially or pre mixed technique. Parturient in the S- group (sequential) had received 10 mg hyperbaric bupivacaine followed by 25 μg fentanyl without barbotage while, in the M − group (premixed) parturient who had received spinal anesthesia using 10 mg hyperbaric bupivacaine 0.5% premixed with 25 μg fentanyl in the same syringe. Then the parturient was asked to lie down supine position with 15-degree lateral to the left immediately after SA has given. Immediately after spinal anesthesia, the onset of sensory block and maximum level of sensory block was assessed by pinching with toothed pick up and recorded. Motor blockade of the lower limbs was tested and scored according to the modified Bromage scale. Time of onset of motor blockade, duration of motor blockade. Bradycardia is managed with atropine while hypotension is treated with crystalloid and if there is not response, adrenaline up to 10ug is administered as final option since there ae no other vasopressor in the hospital. Moreover, shivering is treated with 25 mg IV tramadol, pruritus is also treated with hydrocortisone 50–100 mg IV.

### 2.8 Data collection methods

One of the data collectors attended the operation theater after spinal anesthesia who didn't know the type of spinal anesthesia to record the sociodemographic variables, vital sign, sensory and motor and other intraoperative variables based on the questionnaire. While the other data collector was responsible for data collection in collaboration with the responsible midwives in the postoperative period.

Vital sign was recorded until 4hr. Similarly, pain was assessed by visual analogue scale postoperatively at 30 min, 1 h, 2 h, 3 h, 4 h, 5 h, 6 h, 12 h and 24hr. Rescue analgesia was administered to the patient when VAS≥ 3 cm by the responsible midwives. Most common post-operative analgesics were tramadol (IV) and Diclofenac (IM) to manage moderate to severe pain. The time of first analgesic request was documented. Any complication associated with spinal anesthesia was recorded and managed accordingly with the responsible midwives.

The hemodynamic change after SA and level of post-operative pain were the primary outcomes while, 1st analgesia request time, total analgesic consumption, sensory and motor block characters and complications such as pruritus, shivering, nausea and vomiting, bradycardia and respiratory depression were the secondary outcomes.

### 2.9 Data quality control and assurance

To assure quality of data, training on the objectives, relevance of the study and brief orientations on the assessment tools was given for data collectors and supervisors. In addition to this, pretest of the data collection tool (questionnaire) was done by taking 5% of the total sample size at the referral hospital 2 weeks before the start of the main study, which were not included in the main study. The data were checked for completeness, accuracy and clarity on each day of collection by the principal investigator. The data cleanup and cross checking; arranging materials sequentially and keeping in a safe and secure place was done before analysis.

### 2.10 Data analysis and interpretation

The data were checked manually for completeness and was cleaned and entered into Epi Info version 7.0 and exported into SPSS Version 22 for analysis. A Shapiro Wilk test was used to test for distributions of data while homogeneity of variance between the groups was assessed using Levene's test. The data were normally distributed and homogenous with exception of postoperative pain severity, post-operative analgesia consumption, level and onset of sensory block. Data was statistically described in terms of mean ± SD for independent *t*-test result, median (IQR) for man Whitney *U* test result and frequencies for chi square test results. Chi square test was employed to compare for categorical variables. P value < 0.05 was considered as statistically significant.

## 3 Result

### 3.1 Demographic and perioperative characteristics of the study participants

A total of 66 patients (33 pregnant mothers in each group) was involved in analysis of the study. There was no statistically significant difference between two groups in demographic data and intra operative characteristics (see Table 1).

**Table 1:** Demographic data and intra-operative Characteristics of the parturient who underwent CS.

Variables		M group	S group	P value
Age(year)		27 ± 3.969	27.76 ± 4.637	0.629
Weight (k.g)		69.697 ± 5.86	69.758 ± 4.53	0.963
Height(cm)		161 ± 3.26	160.485 ± 4.16	0.492
Parity (%)	< p3	16(48.6%)	17 (51.4%)	0.805
	≥ p3	15 (48.4%)	18 (51.6%)	
BMI (k.g/m^2^)		25.399 ± 1.79	26.104 ± 1.411	0.08
Duration of Surgery (min)		47.36 ± 7.508	46.64 ± 7.937	0.703
Blood loss in ml		343.64 ± 61.483	365.45 ± 41.615	0.096
Intra operative fluid(ml)		1772.73 ± 264.89	1606.06 ± 280.557	0.16

NB: *Result presented as Mean ± SD, S- sequential group, M – pre mixed group, cm = centimeter = kilogram, m*^*2*^*= meter square, min = minute, MI = body mass index, p = parity, ml = milliliter and SD-standard deviation: p value < 0.05 was considered as significan*t.

### 3.2 Comparison of intra and post-operative mean atrial blood pressure at different time interval

**Fig. 2:**
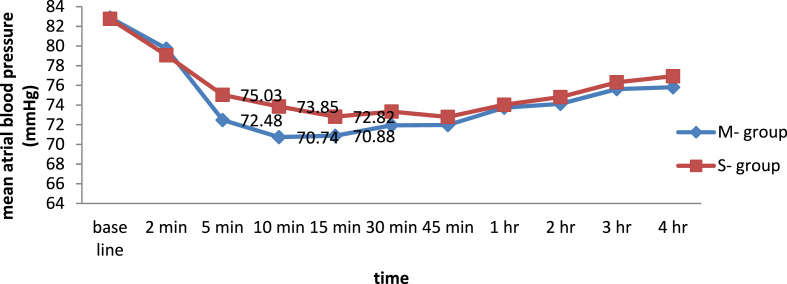
Line graph showing the mean arterial blood pressure at different time interval between M − and S- group undergoing elective cesarean section.

The result shows that there was a significant difference in intra-operative and post-operative mean arterial blood pressure at 5 min, 10 min and 15 min (p < 0.05) but there was no significance difference at 30 min, 45 min, 1hr, 2 h, 3hr, and 4 h, (p > 0.05) (See Fig. 2).

### 3.3 Comparison of intra and post-operative mean heart rate

**Fig. 3:**
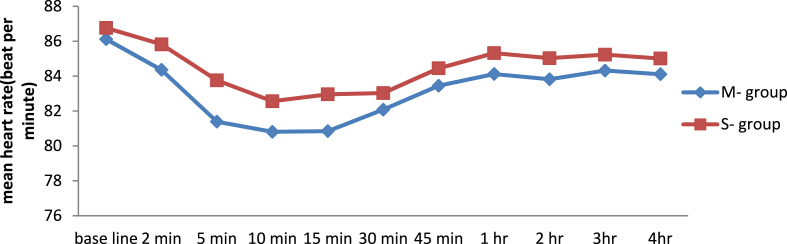
Line graph showing the mean heart rate at different time interval between M − and S- group undergoing elective cesarean section.

There was non-significant difference in the intra-operative and post-operative mean heart rate between the two groups at baseline, 2 min, 5 min, 10 min, 15 min, 30 min, 45 min, 1 h, 2 h, 3hr, and 4 h, p value (p > 0.005 (See Fig. 3).

### 3.4 Comparison of postoperative pain severity by VAS scale

The result was comparable at 30 min, 1hr, 2 h, 3 h, 12 h and 24 h. But the median pain score was significantly lower in the S- group than M-group at 4th hr, at 5th hr and at 6th hr, (p < 0.05) (Table 2).

**Table 2:** Comparison of post-operative pain VAS score between M and S groups in patients undergoing elective cesarean section.

Time	M − group	S- group	P - value
VAS at 30 min	0	0	1.00
VAS at 1hr	0	0	1.00
VAS at 2hr	0	0	1.00
VAS at 3hr	0	0	1.00
VAS at 4hr	3(2–3)	2.5(2–3)	0.045
VAS at 5hr	5.5(5–6)	5(4.5–5.5)	0.005
VAS at 6hr	5.5(4.5–5.75)	5(4.25–5.5)	0.036
VAS at 12 h	5(4–5)	5(4–4.5)	0.957
VAS at 24 h	3(2.5–3)	3(2–3)	0.426

Abbreviation: hr, hour: VAS, visual analogues scale.

### 3.5 Comparison of first rescue analgesic request time and post-operative consumption of analgesia between groups

The mean time to seek 1st request of analgesia was prolonged in S group compared with M group. Furthermore, median total tramadol consumption was lower in S group, but total Diclofenac consumption was not significantly different between groups (see Table 3).

**Table 3:** Showing first rescue analgesic request time and consumption of analgesia between M − group and S- group in patients who underwent elective cesarean section under spinal anesthesia.

Variables		M − group	S- group	P value
First rescue analgesic request time		(261.39 ± 25.378) *	(287.909 ± 15.255) *	<0.001
Total analgesic consumption	Tramadol (IV)	50(50–75)**	50(50)**	0.047
	Diclofenac (IM)	75(0–75)**	75(0–75)**	0.775

NB: Result presented as * = M ± SD in minutes, ** = median (IQR), IM = intramuscular, IV = intravenous, p < 0.05 was considered as statically significant.

### 3.6 Characteristics of block

There was no significant difference on maximum level of sensory block between the two groups, (p > 0.05) but regarding the duration of motor block there was statically significant difference between the groups. (See Table 4).

**Table 4:** Comparison of block character between the two groups who undergone elective Cesarean Section under spinal anesthesia.

Variables	M-group	S- group	P value
Time taken for onset of sensory block	4(4–5)*	4(3–4)*	0.001
Time taken for onset of motor block	5(4.5–6)*	5(4–5)*	0.036
Maximum level of sensory block	T5(T5-T6)*	T6(T5-T7)*	0.06
Sensory regression	and 81.5 ± 8.6	88.3 ± 13.7	0.02
Duration of motor block	(240.27 ± 14.116) **	(255.64 ± 12.874)**	0.001

NB: Values, *: median (IQR) in minute, **: mean ± SD in minute, where, IQR = interquartile range and SD = standard deviation, T = Thoracic dermatome, p value < 0.05 was considered as statistically significant.

### 3.7 Comparisons of incidence of intraoperative complication between the two groups

**Fig. 4:**
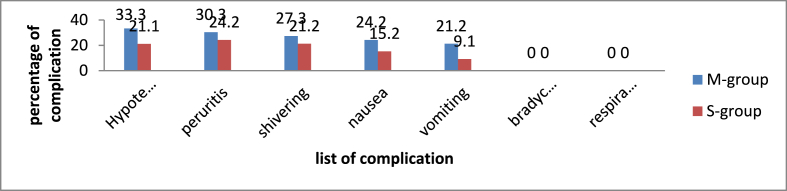
Bar graph showing comparisons of incidence of intra operative complication between S-group and M − group of patients undergoing elective cesarean section.

The Chi square test result shows that the incidence of hypotension between the group was 33.3% in M –group and 21.1% in S- group, (x^2^ = 4.227, p = 0.04). Chi square test result for, nausea, vomiting, shivering and pruritus were comparable between the two groups (p > 0.05), but there was no incidence of bradycardia and respiratory depression (See Fig. 4).

## 4 Discussion

This study was conducted to find the hemodynamic and analgesic effects after administration of sequential and premixed use of hyperbaric bupivacaine and fentanyl of parturient undergoing elective cesarean section under spinal anesthesia.

Multiple trials have been conducted to increase to quality of spinal anesthesia by adding clonidine, dexmedetomidine to bupivacaine in premixing and with separate syringe, they conclude that separate adjuvants with bupivacaine have better hemodynamic and analgesic outcome [18,24] which are in line with this result.

In this study, we found that there was significant reduction in intra operative mean arterial blood pressure at 5th, 10th and 15th min in the M − group compared to the S- group. But there was no significant difference between the groups at 30th min, 45th min, 1st, 2nd, 3rd, and 4th hr after spinal anesthesia which is in line with another study [25]. A similar result was reported by Amraly and his colleagues showed that early hypotension occurred in group M as compared to group S and also found that frequency of drop in mean arterial blood pressure in group S was lower as compared to the group M(p < 0.05) [5]. In contrast to this, another study concluded that there was no significant difference in each mean arterial measurement time between premixing and Sequential opioid-bupivacaine groups [20]. This might be due the previous study added morphine with fentanyl and bupivacaine which might alter the property of drugs.

The present study demonstrated that there was no statically significant difference on intra and post-operative mean heart rate at different time interval between S- group and M − groups. This might be due to; in both groups the level of sympathetic blockage does not reach to the cardio accelerator nerves (T1-T4). In this study, the median time for the onset of sensory and motor block was significantly lower in the S- group compared to the M-group, which is supported by another RCT [26]. However, there was significant difference on the median of the maximum level of sensory block between M-group and S-group T5 (T5-T6) vs T6 (T5-T7) respectively.(p = 0.06) which is in line with another study stating that time to highest level of both sensory and motor was significantly high in M group compared with S group [25]. However, another studies conclude that there was no significant difference between groups [21,27].

With regard to this study, the severity of pain VAS scores was comparable at 30 min, 1hr, 2 h and 3hr between M − and S- group after cesarean section. This might be due to the analgesic effect of spinal anesthesia that continue in both M and S group [28]. But there was statically significant difference in median VAS score at 4th, 5th and 6th hr. this result is in line with another RCT conducted in patients with lower limb orthopedic surgery [29]. The reason might be separate injection of fentanyl and hyper baric bupivacaine allows fentanyl to work on its own maximum effect in the spinal cord preventing visceral pain. However, after 6 h there was no significant difference in median VAS scores at 12 h and 24 h postoperatively this might be due to the duration of intrathecal fentanyl is expected to stay around 6hr and both groups were treated with analgesic drugs.

Other study showed that total analgesic consumption was comparable between M and S groups [21]. In contrast, the current result showed that there was a significantly higher Tramadol consumption in M group as compared with S group. But comparable consumption of Diclofenac. This difference might be due to prolonged first rescue analgesic request time and improved in quality of block in S-group compared to M − group. the current study is supported by another study concluded that total morphine consumption was higher in M groups as compared with S group [20].

Regarding the incidence of intra and post-operative complication, this study showed that hypotension was encountered in 11(33.3%) patients in M − group and 3(21.1%) in S-groups (x^2^ = 4.227, p = 0.040). The hypotension was managed with IV fluid. None of the parturient needed Vaso-active drugs. In line with our result a randomize control trial study done by Amr Aly et al. on 60 parturient who undergone elective cesarean section under spinal anesthesia demonstrated that the incidence of hypotension was 32 (51.6%) in Group M and 18(29%) in Group S (*x*^*2*^ = 6.56, *P* < 0.05) [5]. The reason for this might be the mixture of hypobaric fentanyl and hyperbaric bupivacaine sank down, then they crept up together when the patient lay down acting synchronously on the same level.

There was other intra and post-operative complication like pruritu, shivering, nausea and vomiting without statically significant difference between the two groups. There was no incidence of respiratory depression and bradycardia. This is in line with another randomized control trial [26,30].

## 5 Strength and limitation of study

Besides homogeneity, it was observational, not a randomized control trial. It was difficult to control the speed of intrathecal injection which was different from anesthetist to anesthetist. We used <90mmhg and <70 mmhg fr hypotension, Due to fear and anxiety of covid 19, parturient might not report clear and representative information and small sample size.

## 6 Conclusion and recommendation

The result of our study demonstrates that the use of sequential administration of intrathecal fentanyl and hyperbaric bupivacaine for patients undergoing elective cesarean section under spinal anesthesia has better hemodynamic stability, improve the quality of anesthesia and increases the time for first rescue analgesic request time. We suggest using sequential administration of fentanyl with hyperbaric bupivacaine to have good hemodynamic stability and quality of analgesia for patients undergoing elective cesarean section.

## Ethical approval

Ethical clearance was obtained from the department ethical clearance committee of Addis Ababa Health Bureau.

## Sources of funding

Hiwot fana comprehensive referral hospital for data collection.

## Author contribution

Please specify the contribution of each author to the paper, e.g. study design, data collections, data analysis, and writing. Others, who have contributed in other ways should be listed as contributors.

## Research registration

Researchregistry7457

https://www.researchregistry.com/browse-the-registry#home/

## Guarantor

Habtu Adane Aytolign.

## Funding

Hiwot fana comprehensive referral hospital for data collection.

## Data availability

All data generated or analyzed during this study are included in this article and found on reasonable base.

## Provenance and peer review

Not commissioned, externally peer-reviewed.

## Declaration of competing interest

The authors declare that they have no conflict of interest on the publication of this research.

## List of acronyms and abbreviations

ASA: American society of anesthesiology
BMI: Body mass index
Cm: Centimeter
CS: Cesarean section
CSF: cerebiro spinal fluid
ETB: Ethiopian birr
HB: hyper baric bupivacaine
HR: Heart rate
Hr: hour
IQR: interquartile range
K.G: kilogram
M: pre mixed group
MAP: mean arterial blood pressure
Min: minutes
Mg: milligram
S: Sequential group
SA: Spinal anesthésia
SD: standard déviation
SPSS: Statistical Package for Social Sciences
VAS: visual analogue scale
